# Novel candidate factors predicting the effect of S-1 adjuvant chemotherapy of pancreatic cancer

**DOI:** 10.1038/s41598-021-86099-0

**Published:** 2021-03-22

**Authors:** Katsutaka Mitachi, Kyohei Ariake, Hiroki Shima, Satoko Sato, Takayuki Miura, Shimpei Maeda, Masaharu Ishida, Masamichi Mizuma, Hideo Ohtsuka, Takashi Kamei, Kazuhiko Igarashi, Michiaki Unno

**Affiliations:** 1 Department of Surgery, Tohoku University Graduate School of Medicine, Sendai, Japan; 2 Department of Biochemistry, Tohoku University Graduate School of Medicine, Sendai, Japan; 3 Department of Pathology, Tohoku University Hospital, Sendai, Japan

**Keywords:** Cancer, Gastroenterology, Oncology

## Abstract

The collagen gel droplet-embedded drug sensitivity test (CD-DST) was revealed to be useful for predicting the effect of S-1 adjuvant chemotherapy for pancreatic ductal adenocarcinoma (PDAC). However, collection of an adequate number of PDAC cells is difficult due to the surrounding fibroblasts. Thus, the aim of this study was to discover novel biomarkers to predict chemosensitivity based on the CD-DST results. Proteomics analysis was performed using liquid chromatography tandem mass spectrometry (LC–MS/MS). Candidate proteins were validated in patients with 5-FU CD-DST results via immunohistochemistry (IHC). The relationships between the candidate proteins and the effect of the adjuvant S-1 were investigated via IHC. Among the 2696 proteins extracted by LC–MS/MS, C1TC and SAHH could accurately predict the CD-DST results. Recurrence-free survival (RFS) was significantly improved in the IHC-positive group compared with the IHC-negative group in both factors. The negative group did not show a significant difference from the group that did not receive S-1. The double-positive group was associated with significantly prolonged RFS compared to the no adjuvant chemotherapy group. C1TC and SAHH have been shown to be useful biomarkers for predicting 5-FU sensitivity as a substitute for the CD-DST in adjuvant chemotherapy for PDAC.

## Introduction

The prognosis for patients with pancreatic ductal adenocarcinoma (PDAC) is extremely poor^1^. The postoperative 5-year survival rate is less than 10% due to the high incidence of postoperative recurrence^2^. S-1 adjuvant chemotherapy is strongly recommended for PDAC in Japan, considering the result of the JASPAC-01 study, which demonstrated an improvement in postoperative overall survival compared with gemcitabine (GEM) monotherapy^3^. S-1 combines tegafur, a 5-FU prodrug, gimeracil, a biochemical modulator of 5-FU, and oteracil potassium. Thus, S-1 potentiates the effect of 5-FU. 5-FU has been widely used in chemotherapy for gastrointestinal cancers, including PDAC^4^. 5-FU is a key drug for PDAC treatment, as 5-FU-based chemoradiotherapy is used for unresectable locally advanced (UR-LA) PDAC^5^, and FOLFIRINOX therapy is used for unresectable metastatic (UR-M) PDAC^6^. However, some patients show poor sensitivity to 5-FU. These patients do not benefit from chemotherapy and gain only the adverse effects of anticancer drugs. Thus, the prediction of chemosensitivity is required before initial treatment.

Several papers have described chemosensitivity in PDAC^4,7,8^, but none have been clinically applied. The collagen gel droplet-embedded drug sensitivity test (CD-DST), which is an anticancer drug sensitivity test, has been shown to be useful in several cancers^9–11^. The CD-DST was performed to assess in vitro sensitivity. Briefly, fresh surgical specimens from tumors were collected aseptically. The samples were digested with a cell dispersion enzyme solution. After preliminary culturing, the cells were added to collagen gel droplets, and anticancer drugs were added. After contact for 2 h, the cells were cultured in serum-free medium for 7 days. The control group was not exposed to the anticancer drug. Sensitivity was assessed by the *T/C* ratio, calculated as the ratio of the number of cancer cells in the treatment group (T) to the number of cancer cells in the control group (C). A *T/C* ratio < 50% was considered high sensitivity, and a *T/C* ratio > 60% was considered low sensitivity^12^. The CD-DST made it possible to predict the effect of 5-FU in gastric cancer, colon cancer, and ovarian cancer patients^9,11,13^. Previously, we elucidated that the CD-DST is useful for predicting the effect of postoperative adjuvant therapy with S-1 in resected PDAC cases^10^. However, due to the large amount of interstitium in the tissue, it was difficult to collect the required number of samples for this test, and the CD-DST results were obtained from only 53.8% of cases. Therefore, it is necessary to search for new biomarkers that can predict sensitivity by utilizing the results of the CD-DST.

The purpose of the present study was to identify a novel biomarker that usefully reflects the sensitivity of PDAC to 5-FU and can be substituted for the CD-DST to predict the effect of S-1 adjuvant chemotherapy.

## Results

### Step 1: extraction stage and discovery stage

**Figure 1:**
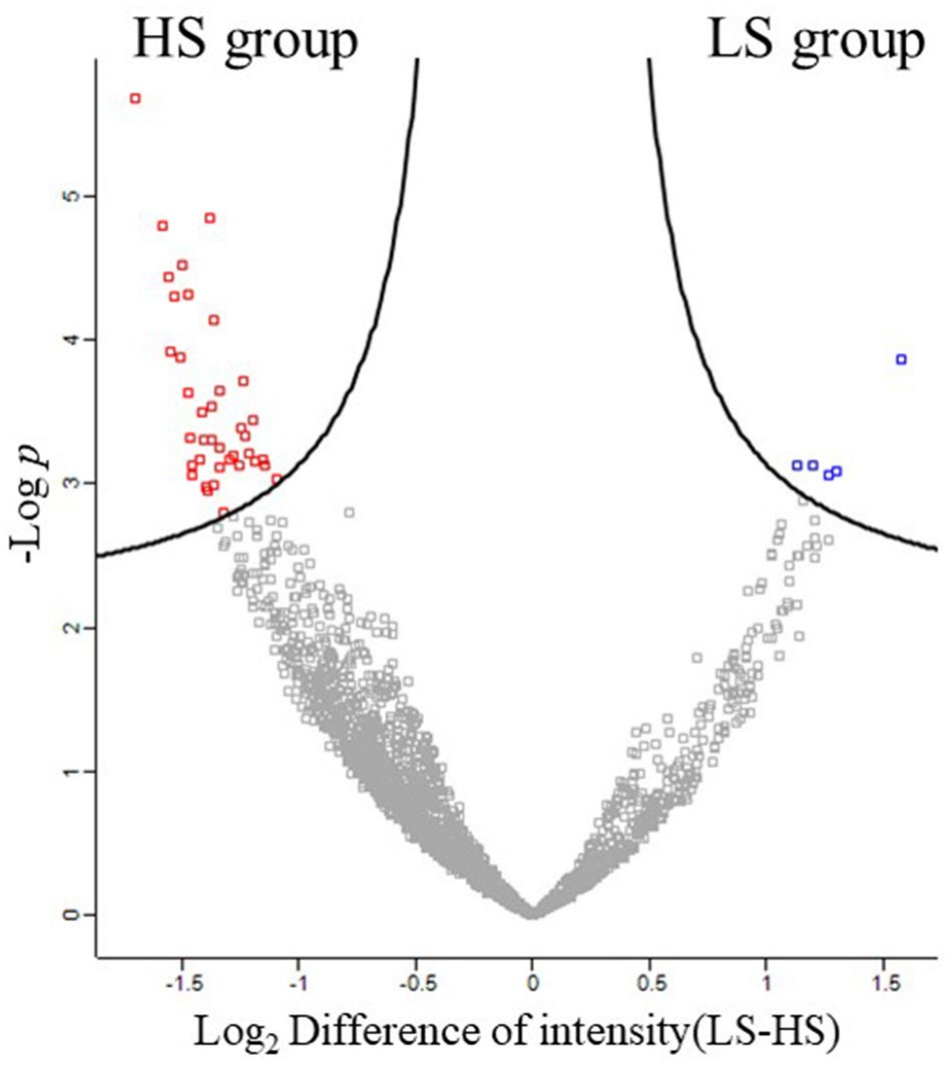
Volcano plot of detection protein created by Perseus. The difference in the intensity of each protein between the HS group and the LS group was calculated by Perseus, and significant difference was evaluated by Student's t-test to prepare a Volcano plot. The analysis was performed with fold change > 0.1 and FDR = 0.05. HS high sensitivity, LS low sensitivity.

The top 5 patients with high sensitivity (HS) and low sensitivity (LS) considering 5-FU sensitivity evaluated by the CD-DST were selected. As shown in Supplementary Table S1 online, there was no statistically significant difference in background characteristics between the two groups. Cancer cells were selectively harvested from formalin-fixed paraffin-embedded (FFPE) tissues using laser microdissection (LMD). Protein was extracted via global shotgun proteomics using liquid chromatography tandem mass spectrometry (LC–MS/MS)-based proteomics. Analysis of MS data with MaxQuant yielded 2696 proteins. Perseus analysis of the MaxQuant output data revealed significant differences in approximately 5 proteins in the LS group and 39 proteins in the HS group (Fig. 1).

The functions of each candidate protein were examined through a PubMed online search using the keywords of each protein name to evaluate whether these proteins are correlated with chemosensitivity or drug resistance. As a result, 10 proteins, namely, LAMA4, TGM2, SAHH, BRI3B, OTUB1, C1TC, F10A1, TRAP1, NDKA, and F16P1^14–28^, were extracted.

Next, immunohistochemistry (IHC) analysis was performed to determine whether these factors could well predict the results of the CD-DST. Among the ten factors, the expression of C1TC was significantly elevated in the HS group (p = 0.026), and SAHH also tended to show elevated expression in the HS group (p = 0.052). Thus, these two factors were used to estimate 5-FU sensitivity evaluated by the CD-DST (Table 1), and further analysis was conducted in the next step, the clinical stage.

**Table 1:**
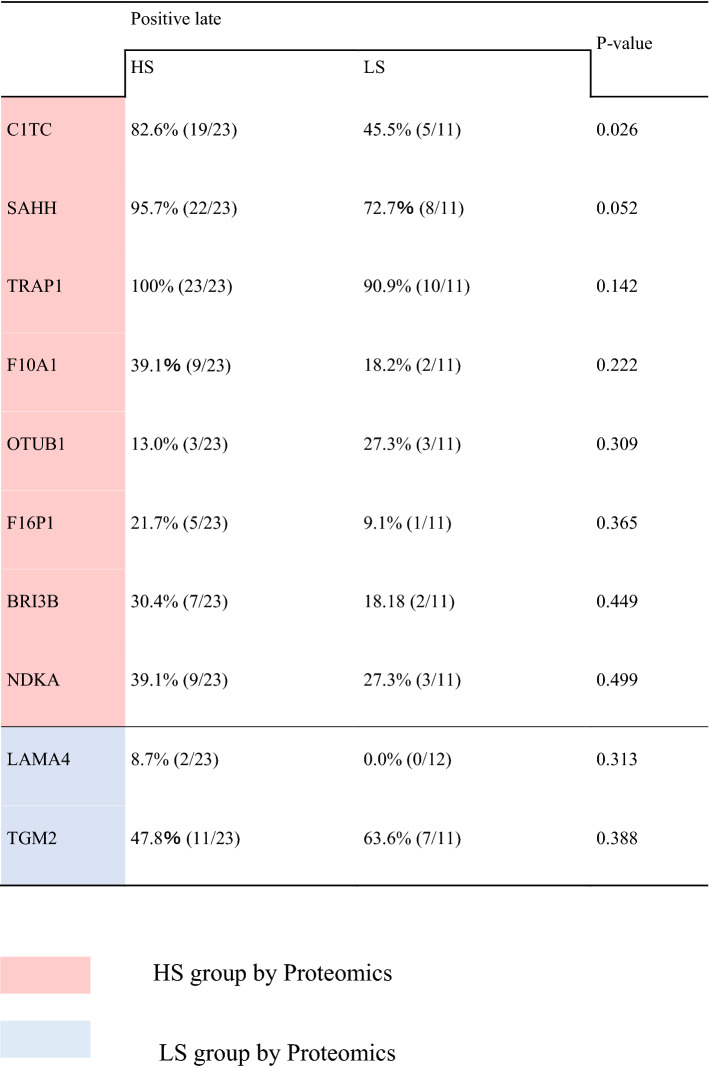
Clinicopathological background of LC–MS/MS cohort.

### Step 2: clinical stage

To confirm the clinical utility of C1TC and SAHH, IHC analysis was performed targeting 49 patients who received S-1 adjuvant chemotherapy after curative resection for PDAC. As a control cohort, we analyzed 35 patients who did not receive adjuvant chemotherapy after surgical resection. Among the 49 patients, 31 were considered positive and 18 were considered negative for C1TC. SAHH was also considered as positive in 31 patients and negative in 18 patients. There was no significant difference in background characteristics between patients who were positive or negative for either C1TC or SAHH (Table 2).

**Table 2:** Patient clinicopathological characteristics of positive and negative group in C1TC and SAHH.

		C1TC			SAHH		
		Positive n = 31	Negative n = 18	P value	Positive n = 31	Negative n = 18	P value
Age (years)	Median (range)	65 (47–81)	68 (44–77)	0.424	66 (44–79)	65 (48–81)	0.618
Sex	Male:female	18:13	10: 8	0.864	19:12	9:9	0.442
CA19-9 (U/mL)	Median (range)	40.5 (0.6–1018)	62.6 (1.5–890.3)	0.494	48.5 (0.6–649.4)	29.5 (1.6–1018)	0.604
Resectability classification	R:BR	22:9	16:2	0.131	25:6	13:5	0.500
Location of the tumor	Head:body-tail	18:13	9:9	0.585	18:13	9:9	0.585
Tumor size (mm)	Median (range)	25 (0–42)	25 (4–35)	0.853	25 (0–42)	25 (4–40)	0.526
Anterior serosal invasion	Positive:negative	22:9	15:3	0.322	23:8	14:4	0.778
Retroperitoneal invasion	Positive:negative	24:7	14:4	0.977	24:7	14:4	0.977
Portal vein invasion	Positive:negative	6:25	4:14	0.811	5:26	5:13	0.336
Lymph node metastasis	Positive:negative	15:16	13:5	0.100	19:12	9:9	0.442
Residual cancer (R1)	R0:R1	28:3	16:2	0.874	28:3	16:2	0.874

**Figure 2:**
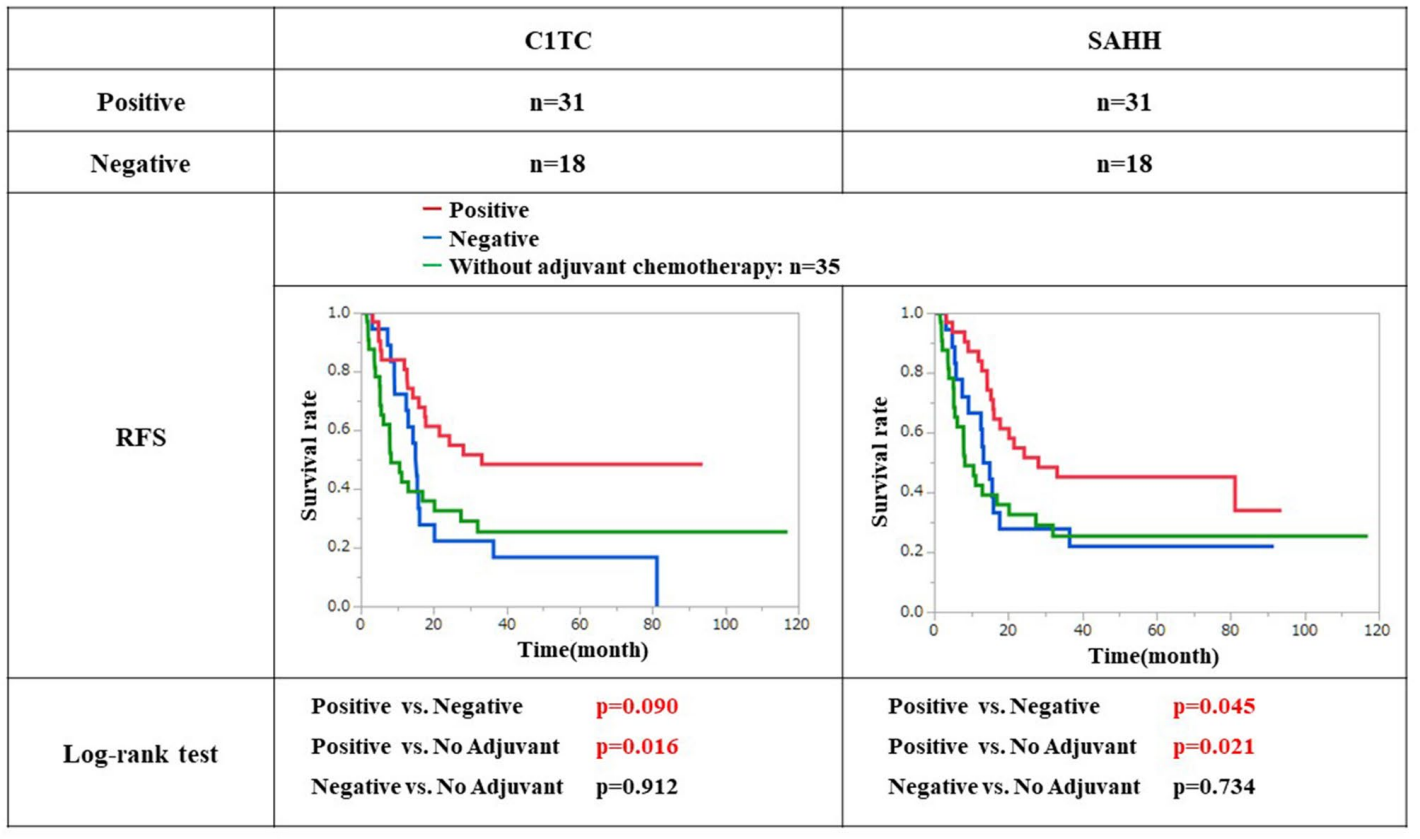
Recurrence free survival of patients with expressing each candidate factors and no adjuvant chemotherapy. In both C1TC and SAHH, a significant improvement of RFS was observed in the positive group compared to the negative group. Furthermore, positive group showed significant improvement of RFS compared with no adjuvant chemotherapy group. On the other hands, no significant difference was fond between negative group and no adjuvant group in both C1TC and SAHH.

A comparison of recurrence-free survival (RFS) between C1TC-positive and C1TC-negative patients showed significantly improved RFS in C1TC-positive patients compared with C1TC-negative patients (p = 0.009) (Fig. 2). Similar results were also obtained for SAHH, as RFS was prolonged in SAHH-positive patients (p = 0.045) (Fig. 2). RFS in these patients was also compared with that in 35 patients who did not receive adjuvant treatment. Among the C1TC-positive patients, those who received adjuvant treatment had significantly better RFS than those who did not (p = 0.016), and the same results were also obtained from the SAHH-positive group (p = 0.021). On the other hand, no significant difference was found in C1TC-negative patients (p = 0.912) or in SAHH-negative patients (p = 0.734) when the no adjuvant therapy group was compared with the adjuvant therapy group.

**Figure 3:**
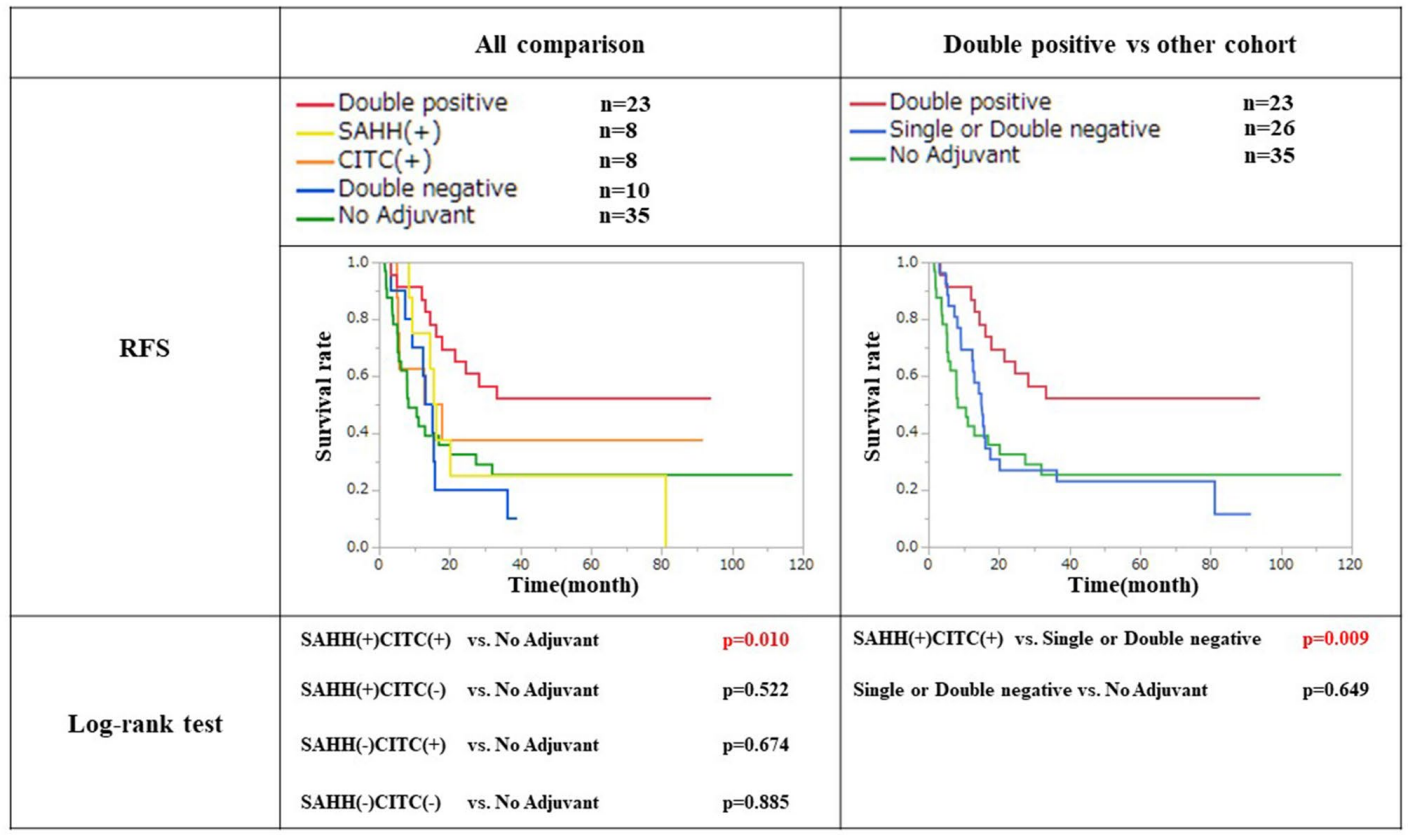
Recurrence free survival after combining C1TC and SAHH. There were 23 cases of both C1TC and SAHH showed positive expression, 8 cases showed both negative expressions, 8 cases showed only SAHH positive and 8 cases of C1TC positive respectively. Double positive group showed a significant improvement of RFS compared to No adjuvant group. On the other hands, when C1TC and/or SAHH was negative, there was no significant difference compared with no adjuvant group. When C1TC and/or SAHH negative cases was combined into one group and compared with double positive or No adjuvant group, RFS was dramatically worse compared with double positive group and almost similar with No adjuvant group.

Next, we conducted an analysis combining C1TC and SAHH. There were 23 patients in the double-positive (DP) group (SAHH(+)C1TC(+)), 8 patients in the SAHH only positive group (SAHH(+)C1TC(−)) and 8 patients in the C1TC only positive group (SAHH(−)C1TC(+)). The other 10 patients were classified as the double negative (DN) group (SAHH(−)C1TC(−)). When the no adjuvant therapy group was added to these four groups and examined again, only the DP group showed significantly prolonged RFS compared to the no adjuvant therapy group (p = 0.009) (Fig. 3). If either or both were negative, no significant difference was found upon comparison with the no adjuvant therapy group (vs SAHH(+)C1TC(+): p = 0.522, vs SAHH(−)C1TC(+): p = 0.674, vs DN: p = 0.885) (Fig. 3). When the single- or double-negative patients were combined into one group and compared with those in the DP or no adjuvant therapy group, RFS was dramatically worse in these groups compared with the DP group (p = 0.009) and similar to the no adjuvant therapy group (p = 0.649). Severe differences in background characteristics were not found among these three groups (see Supplementary Table S2 online).

To elucidate whether C1TC-SAHH expression could be a valuable factor to predict the effect of S-1 adjuvant chemotherapy, we analyzed predictive risk factors for recurrence among 49 patients who received S-1 adjuvant chemotherapy after surgical resection for PDAC. Univariate analysis demonstrated the prognostic value of CA19-9, anterior serosal invasion and C1TC-SAHH double positivity. Multivariate analysis demonstrated that only C1TC-SAHH double positivity was a predictive risk factor for recurrence (hazard ratio [HR]: 0.45, 95% confidence interval [CI] 0.21–0.93; p = 0.030) (Table3).

**Table 3:** Univariate and multivariate analyses of risk factors for recurrence.

	Univariate analysis		Multivariate analysis	
Age (≤ 65 years)	1.57 (0.77–3.39)	0.217		
Sex (male)	0.90 (0.49–1.84)	0.768		
Resectability status (R)	1.05 (0.48–2.62)	0.915		
CA19-9 before initial treatment (≥ 100 U/mL)	2.27 (1.09–4.58)	0.029	1.99 (0.95–4.05)	0.067
Tumor position (ph)	1.74 (0.85–3.76)	0.131		
Tumor size (≥ 20 mm)	1.92 (0.87–4.83)	0.110		
Anterior serosal invasion (positive)	2.86 (1.19–8.47)	0.017	2.39 (0.98–7.14)	0.055
Retroperitoneal invasion (positive)	1.85 (0.77–5.47)	0.179		
Portal vein invasion (positive)	1.62 (0.64–3.60)	0.284		
Lymph node metastasis (positive)	1.54 (0.75–3.31)	0.241		
Residual cancer (R1)	1.73 (0.51–4.46)	0.339		
CITC, SAHH double positive	0.39 (0.18–0.79)	0.009	0.45 (0.21–0.93)	0.030

## Discussion

This is the first study to elucidate factors that could be used to predict sensitivity to S-1 adjuvant chemotherapy in PDAC patients by performing proteomics analysis using clinical specimens. Our results suggest that C1TC and SAHH could well reflect 5-FU sensitivity and serve as predictive risk factors for recurrence after S-1 adjuvant chemotherapy.

PDAC has a high recurrence rate after radical resection and a poor prognosis. Multidisciplinary treatment, including chemotherapy, is required. Based on the results of recent clinical trials, it is recommended to use adjuvant chemotherapy after curative resection. In recent years, the usefulness of neoadjuvant chemotherapy mainly for resectable PDAC has been reported (Prep-02/JSAP-05)^29^. Furthermore, an increasing number of reports have shown improved prognosis by conversion surgery for patients who have successfully received chemotherapy for unresectable PDAC^6^. Therefore, chemotherapy is at the core of therapeutic strategies in PDAC^30^. Treatment regimens of several drugs either alone or in combination are applied for PDAC but are not always effective for all patients. Therefore, the prediction of sensitivity to anticancer drugs is desired when deciding a treatment regimen.

Both C1TC and SAHH were extracted as factors whose expression was significantly increased in the HS group, and in patients in whom these factors were expressed, a significant improvement in RFS was obtained after S-1 adjuvant chemotherapy. On the other hand, it was clarified that in the patients with negative expression, an additional effect of the treatment could not be expected even when compared with the patient group in which adjuvant chemotherapy was not given. In the present study, only two factors were extracted by the comprehensive analysis, so further analysis was performed by combining these two factors. As a result, RFS improvement by S-1 was obtained only when both factors were positive. C1TC and SAHH double positivity could be a predictive factor of a decreased risk of recurrence after S-1 adjuvant chemotherapy. On the other hand, when either of the two factors was negative, the treatment benefit of S-1 was not observed. All these results demonstrate that the expression of both C1TC and SAHH is essential for the benefits of S-1 adjuvant chemotherapy.

The main action of 5-FU in cells is to exert an antitumor effect by suppressing thymidylate synthase (TYMS) and causing impaired DNA synthesis^15^. Folic acid metabolism is closely related to the action of 5-FU^16^. C1TC is an enzyme involved in the synthesis of tetrahydrofolate (THF) and methylene THF (5–10-CH_2_-THF) in the folic acid metabolism pathway, and methylene THF is combined with TYMS together with FdUMP, a metabolite of 5-FU. This ternary complex suppresses TYMS activity, causing DNA damage and leading to cell death. The results of this study suggest that the decreased expression of C1TC could inhibit formation of the ternary complex reducing the synthesis of methylene THF; thus, the ability of 5-FU to suppress TYMS by inhibiting formation of the ternary complex and cell death was prevented. Taken together, these results suggest that decreased C1TC expression reduces the toxic effect of 5-FU^16^, and the effect of S-1 adjuvant chemotherapy is decreased.

SAHH is an enzyme that removes adenosine from S-adenosyl homocysteine (SAH) in the methionine metabolic pathway to produce homocysteine (Hcy). There is a close coreaction between methionine metabolism and folic acid metabolism called one-carbon metabolism^31^. Hcy from methionine metabolism receives a methyl group (–CH_3_) from methyl THF (5-CH_3_-THF) from folic acid metabolism, and methionine and THF are produced. High SAHH expression in pancreatic cancer is expected to increase Hcy, resulting in activation of the THF cycle in folic acid metabolism and in increased susceptibility to 5-FU. It was previously reported that in prostate cancer, the activation of methionine metabolism had a synergistic effect with 5-FU and selectively suppressed TYMS^31^. SAHH has also been reported as a tumor suppressor^32^. Overexpression of SAHH has been shown to induce apoptosis in squamous cell carcinoma of the esophagus^33^. In addition, adenosine produced with Hcy when SAHH degrades SAH has been reported to induce apoptosis^34–36^. It is suggested that SAHH is involved in the action of 5-FU via adenosine in addition to the folic acid metabolic pathway.

Based on the findings described above, C1TC enhances the action of 5-FU directly from the folic acid metabolism pathway, and SAHH indirectly enhances the action of 5-FU from methionine metabolism via folic acid metabolism. In addition, SAHH may promote the effects of 5-FU by inducing metabolism through the production of adenosine.

This study had some limitations. Since only cancer cells were targeted, the action of interstitial factors was not considered. The stroma surrounding the cancer cells is thought to play a role as a defense mechanism that inhibits the penetration of a drug into a deeply located tumor. The proportion of interstitium in pancreatic cancer tissue varies from patient to patient and may affect individual differences in sensitivity. Therefore, even if the S-1 high-sensitivity protein identified herein is highly expressed, there may be cases in which the actual clinical effect is minimal. In addition, the results of this study were based on a single institution, and they likely cannot be applied to multiple centers, suggesting institutional bias.

However, the factor identified in this study is the first factor identified as a biomarker in PDAC that predicts recurrence after S-1 adjuvant chemotherapy. Although excised specimens were used as the samples in this study, if the expression intensity of C1TC and SAHH can be evaluated by IHC using biopsy tissue such as EUS-FNA, it can be used to select a neoadjuvant chemotherapy regimen. It may also be useful in choosing FOLFIRINOX therapy or GEM with nab-paclitaxel therapy in unresectable PDAC. In the future, it will be necessary to conduct clinical trials to confirm whether the expression of C1TC and SAHH could be used to predict the effect of S-1 adjuvant chemotherapy.

In conclusion, SAHH and C1TC were identified as predictors of sensitivity to S-1 adjuvant therapy for PDAC. It is believed that the effect of S-1 after PDAC resection may not be sufficiently obtained in patients in whom the expression of these factors is not observed.

## Methods

### Ethics statement

This nonrandomized, retrospective study was approved by the Institutional Review Board of Tohoku University (Sendai, Japan) in 2018 (approval no. 2018-1-314). The need to collect informed consent was waived due to the retrospective design of this study, and an “opt-out” method was used. The research was conducted in accordance with the principles outlined in the Declaration of Helsinki.

### Study design

**Figure 4:**
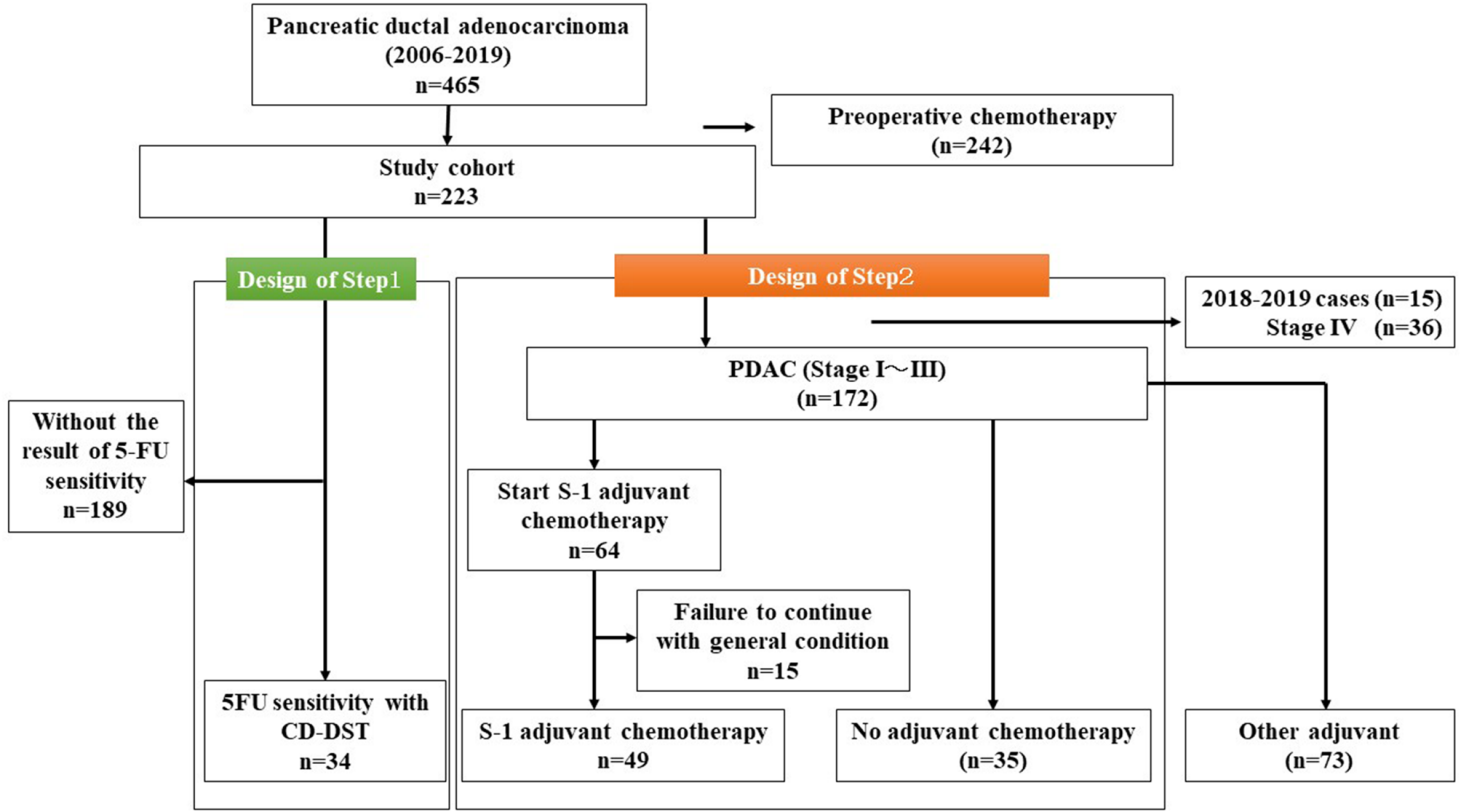
Study design. The cohort of Step1 was targeting the patients evident the results of CD-DST. The cohort of Step2 is targeting the PDAC patients with S-1 adjuvant chemotherapy. We set no adjuvant patients group as control cohort of this study. CD-DST collagen gel droplet embedded drug sensitivity test, PDAC pancreatic ductal adenocarcinoma.

From January 1, 2006, to December 31, 2019, 465 histologically diagnosed PDAC patients were undergoing pancreatectomy in the Department of Surgery at Tohoku University Graduate School of Medicine, Sendai, Japan. A total of 242 patients who received neoadjuvant chemotherapy (NAC) were excluded from this study. Thus, 223 patients were enrolled (Fig. 4).

Step 1: Among 223 patients, 189 without 5-FU sensitivity data measured by the CD-DST were excluded. Among 34 patients, the top 5 with the highest T/C ratio evaluated by the CD-DST were selected as the LS group, and the top 5 with the lowest T/C ratio were selected as the HS group. Samples from these ten patients were subjected to LC–MS/MS analysis. Furthermore, the results of 5-FU sensitivity (according to the CD-DST) were used, and IHC analysis was also performed, targeting these 34 patients to evaluate the candidate factors identified by LC–MS/MS analysis.

Step 2: Among 223 patients, 15 underwent pancreatectomy between 2018 and 2019, and 36 who were diagnosed with stage IV PDAC^37^ were excluded. Thus, 172 patients comprised the cohort for step 2. Sixty-four patients started S-1 adjuvant chemotherapy, and 15 patients could not continue treatment according to their general condition or severe adverse events. Thus, we focused on these 49 patients, including those who received postoperative adjuvant chemotherapy with S-1 for more than half a year and those who experienced recurrence within 6 months during postoperative adjuvant chemotherapy with S-1. Furthermore, 35 patients who did not receive adjuvant chemotherapy were evaluated as the control group.

### Cancer cell collection by LMD

Resected pancreatic cancer specimens were fixed in 4% paraformaldehyde and routinely processed for paraffin sectioning. Ten-micrometer sections were attached to DIRECTORTM slides (Expression Pathology, MD), deparaffinized three times with xylene for 5 min, rehydrated with graded ethanol solutions and distilled water, and then stained with hematoxylin. Cancerous pancreatic ductal cells (10–12 mm^2^) were collected into the cap of a 0.2 ml PCR tube using a Leica LMD7000 (Leica Microsystems GmbH, Wetzler, Germany). The laser setting conditions were based on the method of Longuespée et al.^38^. Peptides were extracted using a Liquid Tissue TM MS Protein Prep Kit (Expression Pathology) according to the manufacturer’s instructions. Briefly, the cellular material, suspended in liquid tissue buffer, was incubated at 95 ℃ for 90 min and then cooled on ice for 3 min. Trypsin was added and incubated overnight at 37 ℃. Dithiothreitol was added to a final concentration of 10 mM, and the samples were heated for 5 min at 95 ℃. The liquid tissue digestate was stored at − 20 ℃ until analysis.

### Shotgun proteomics by LC–MS/MS

The measuring instruments used were an Orbitrap Fusion hybrid mass spectrometer (Thermo Fisher Scientific, USA) and an attached reversed-phase liquid chromatography system (EASY-nLC 1000 HPLC system, Thermo Fisher Scientific). The reversed-phase liquid chromatography system consisted of a C18 PepMap 100 trap column (length 20 mm × inner diameter 75 μm) and a C18 tip column (length 10 cm × inner diameter 75 μm, particle size 3 μm, Nikkyo Technos Co., Ltd.). First, the stored sample was injected into the trap column, washed with a 0.1% formic acid aqueous solution (solvent A), concentrated, and desalted. Then, using acetonitrile (solvent B) containing 0.1% formic acid, elution of the separated peptide by a concentration gradient was carried out for 80 min. At a flow rate of 0.3 μL/min, solvent A: 98% and solvent B: 2% were first changed to solvent A: 72% and solvent B: 28% for 67 min. Furthermore, the mixture was changed to solvent A: 60% and solvent B: 40% for 9 min and finally to solvent A: 5% and solvent B: 95% for 2 min. The eluted peptides were ionized by electrospray and analyzed on a mass spectrometer. General MS conditions were as follows: electrospray voltage, 1.8 kV, no sheath and auxiliary gas flow; capillary temperature, 250 ℃; collision energy (CE), 35%; ion selection threshold 1000 counts for MS/MS, top speed time 4 s, and dynamic exclusion time 60 s.

### Proteomics by label-free quantification (LFQ)

MS data were analyzed using MaxQuant version 1.6.2.10 (Max Planck Institute for Biochemistry, Germany)^39^. Protein N-terminal acetylation and methionine oxidation were set as posttranslational modifications, but no chemical modification was set. The minimum peptide length was set to 7 amino acids. LFQ was enabled, and the LFQ minimum ratio was set to 1. The remaining options were set to default values. The analysis result was output to the text file "ProteinGroups.txt". UniProt KB/Swiss-Prot was used as the reference database.

Protein expression data were statistically analyzed using Perseus version 1.6.2.3 (Max Planck Institute for Biochemistry, Germany)^40,41^. The LFQ intensity was log2 converted, and the Z-score was normalized. Proteins with low expression intensity data were excluded, and missing LFQ values were assigned by random numbers from normal distribution. Hierarchical clustering was performed for log2 conversion LFQ intensity, Student’s t test was performed to examine differences between the HS group and LS group, and the difference in the expression level between the two groups was calculated. A volcano plot was created by calculating the q value, which is the expected value obtained by log-converting the p value. Proteins satisfying a false discovery rate (FDR) = 0.05 and fold change > 0.1 (used as the threshold of the q-value) were judged to be significantly different^42^.

### IHC

**Figure 5:**
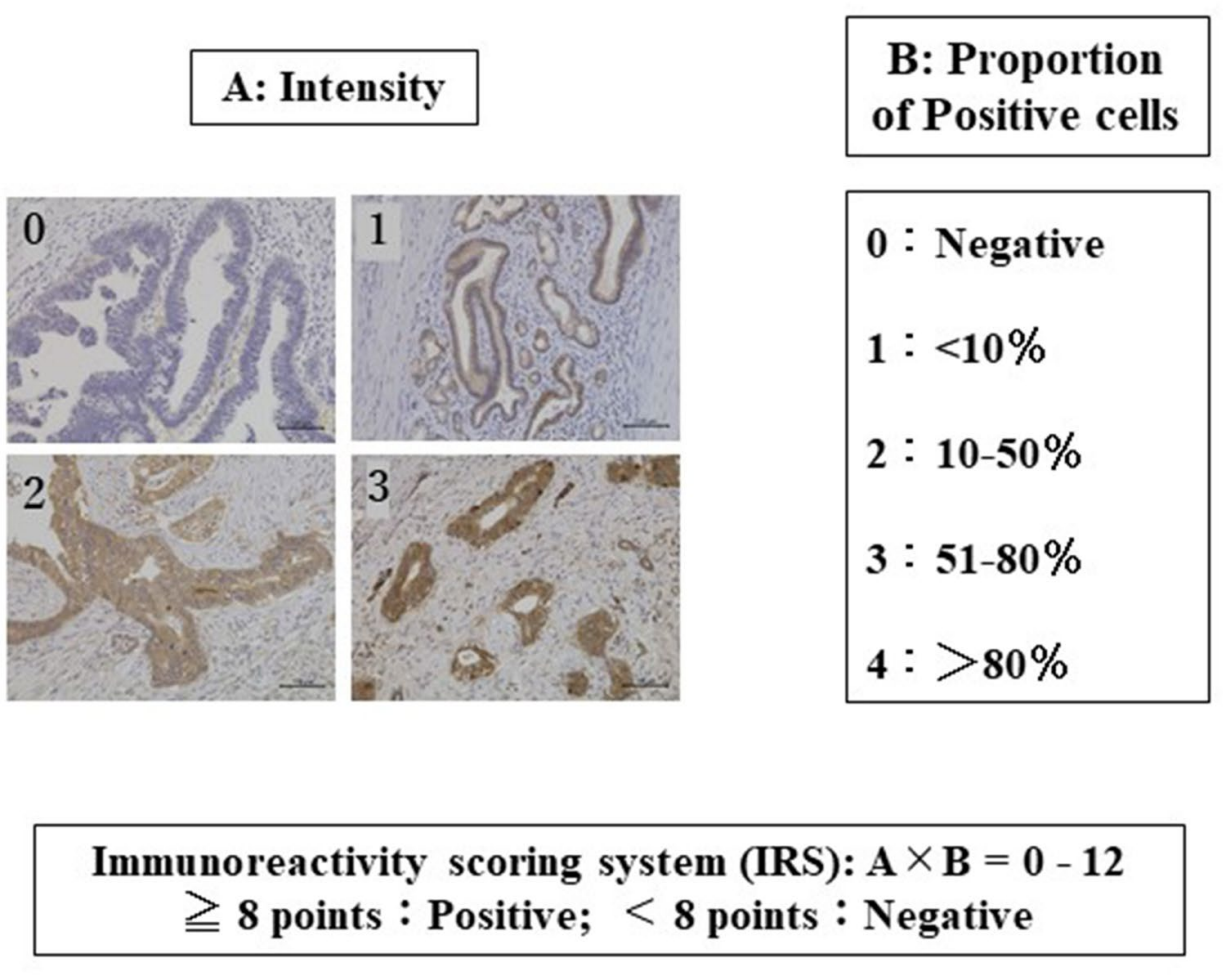
Immunoreactive score (IRS). The Immunoreactivity scoring system (IRS), which is a combination of the intensity (1 to 4 points) and proportion of positive cells, was used. 8 points and more were judged to be IRS positive. Scale bar: 100 μm.

For IHC analysis, 4 μm FFPE tissue sections were deparaffinized with xylene and rehydrated with ethanol solutions and distilled water. Antigen retrieval was performed by heating the sections in 10 mmol/L citrate buffer (pH 6.0) in a microwave oven for 15 min or 120 ℃ for 5 min in an autoclave. Adenosylhomocysteinase (#10757-2-AP, 1:200, Proteintech Group, Chicago, IL, USA), methylenetetrahydrofolate dehydrogenase, cyclohydrolase and formyltetrahydrofolate synthetase 1 (#10794-1-AP, 1:500, Proteintech Group) antibodies were used as the primary antibodies. BRI3 binding protein (**# **PA5-38752, 1:100, Invitrogen by Thermo Fisher Scientific, Waltham, USA), laminin subunit alpha 4 (#10465-1-AP, 1:200, Proteintech Group), transglutaminase 2 (#15100-1-AP, 1:500, Proteintech Group), TNF receptor associated protein 1 (#10325-1-AP, 1:200, Proteintech Group), OTU deubiquitinase, ubiquitin aldehyde binding 1 (#ab198214, 1:200, Abcam), fructose-bisphosphatase 1 (#12842-1-AP, 1:800, Proteintech Group), NME/NM23 nucleoside diphosphate kinase 1 (#3345, 1:100, Cell Signaling Technology, Danvers, USA), and Hsp70 interacting protein (#26581-1-AP, 1:500, Proteintech Group) antibodies were also used. After blocking endogenous peroxidase with methanol containing 0.3% hydrogen peroxidase, the labeled antigens were detected with a horseradish peroxidase EnVision System (DAKO, Glostrup, Denmark) and visualized with 3,30-diaminobenzidine tetrahydrochloride as a chromogen. The sections were lightly counterstained with hematoxylin. Appropriate positive controls were used, in part with reference to the Human Protein Atlas (http://www.proteinatlas.org/). The expression analysis described in step 1 was preliminarily evaluated by two researchers using BZ-9000 and an observation application (Keyence, Osaka) to narrow down the candidate factors. All the evaluations described in step 2 were evaluated by a pathologist in addition to the researchers. The immunoreactivity scoring system (IRS) was used as the criterion^43^ (Fig. 5). The IRS cutoff value was calculated from the receiver operating characteristic (ROC) curve with postoperative recurrence as the outcome in step 2, and a positive score was IRS 8 points or higher in both C1TC and SAHH (C1TC: AUC 0.665, SAHH: AUC 0.609).

### Statistical analysis

All data were collected on January 1, 2021, and entered into a Microsoft Excel spreadsheet (Redmond, WA, USA). All statistical analyses were performed using JMP software, version 14 (SAS Institute Inc., Cary, NC, USA). ROC curve analysis was used to determine the optimal cutoff ratio of IRS for candidate factor extraction. The accuracy of the predictive score was calculated in terms of the sensitivity and specificity of the cutoff ratio. The differences in categorical data between groups were evaluated using Pearson’s chi-squared tests. Continuous variables were compared using the nonparametric Mann–Whitney U test. Survival curves were estimated using the Kaplan–Meier method, and comparisons were performed with the log-rank test. A Cox proportional hazards regression model was used for the multivariate analysis to evaluate the factors associated with recurrence. p < 0.05 was considered statistically significant.

## Supplementary Information

Supplementary Information.
