# Supplementary material for: Novel candidate factors predicting the effect of S-1 adjuvant chemotherapy of pancreatic cancer
Source: Sci Rep. 2021 Mar 22;11:6541. doi: 10.1038/s41598-021-86099-0 (PMC7985196; doi:10.1038/s41598-021-86099-0)
Supplement: Supplementary file 1 — Supplementary Information. [file 41598_2021_86099_MOESM1_ESM.pdf]

# Title: Novel candidate factors predicting the effect of S-1 adjuvant chemotherapy of pancreatic cancer

**Katsutaka Mitachi[1)], Kyohei Ariake[1)], Hiroki Shima[2)], Satoko Sato[3)], Takayuki Miura[1)], Shimpei Maeda[1)], Masaharu Ishida[1)], Masamichi Mizuma[1)], Hideo Ohtsuka[1)], Takashi Kamei[1)], Kazuhiko Igarashi[2)], Michiaki Unno[1)]**

**1)** Department of Surgery, Tohoku University Graduate School of Medicine

**2)** Department of Biochemistry, Tohoku University Graduate School of Medicine

**3)** Department of Pathology, Tohoku University Hospital

**Supplementary Table S1**

Patient clinicopathological characteristics of LC-MS/MS cohort

| | | Low sensitivity n=5 | High sensitivity n=5 | P value |
|---|---|---|---|---|
| **T/C ratio** | **Median (range)** | **100 (94.2-100)** | **37.5 (26.3-43.6)** | **0.003** |
| **Age (years)** | **Median (range)** | **70 (62-79)** | **74 (60-77)** | **0.072** |
| **Sex** | **male：female** | **2 : 3** | **1 : 4** | **0.490** |
| **CA19-9 (U/mL)** | **Median (range)** | **41.3 (1.5-628.1)** | **63.4 (9.1-10239)** | **0.549** |
| **Resectability classification** | **R：BR** | **4 : 1** | **5 : 0** | **0.292** |
| **Location of the tumor** | **Head：Body-tail** | **3 : 2** | **4 : 1** | **0.490** |
| **Tumor size (mm)** | **Median (range)** | **25 (14-35)** | **31 (11-40)** | **0.549** |
| **Anterior serosal invasion** | **positive：negative** | **3 : 2** | **4 : 1** | **0.490** |
| **Retroperitoneal invasion** | **positive：negative** | **3 : 2** | **4 : 1** | **0.490** |
| **Portal vein invasion** | **positive：negative** | **1 : 4** | **0 : 5** | **0.292** |
| **Lymohnod metastasis** | **positive：negative** | **3 : 2** | **3 : 2** | **1.000** |
| **Residual cancer (R1)** | **R0 : R1** | **5：0** | **3：2** | **0.114** |

**Supplementary Table S2**

Patient clinicopathological characteristics of three groups as Double positive group, Single or Double negative group and No adjuvant group.

| | | Double Positive n=23 | Single or Double negative n=26 | No adjuvant n=35 | P value |
|---|---|---|---|---|---|
| Age (years) | Median (range) | 66 (47-79) | 66.5 (44-81) | 76 (51-88) | <0.001 |
| Sex | male：female | 12 : 11 | 16 : 10 | 24 : 11 | 0.454 |
| CA19-9 (U/mL) | Median (range) | 43.7 (0.6-649.4) | 148.5 (2-10239) | 39.7 (1.5-1018) | 0.066 |
| Resectability classification | R：BR | 17 : 6 | 21 : 5 | 25 : 10 | 0.693 |
| Location of the tumor | Head：Body-tail | 12 : 11 | 15 : 11 | 22 : 13 | 0.720 |
| Tumor size (mm) | Median (range) | 24 (0-42) | 26 (0-80) | 25 (4-40) | 0.217 |
| Anterior serosal invasion | positive：negative | 15 : 8 | 22 : 4 | 17 : 18 | 0.011 |
| Retroperitoneal invasion | positive：negative | 17 : 6 | 21 : 5 | 26 : 9 | 0.799 |
| Portal vein invasion | positive：negative | 4 : 19 | 6 : 20 | 12 : 23 | 0.323 |
| Lymohnod metastasis | positive：negative | 13 : 10 | 15 : 11 | 20 : 15 | 0.997 |
| Residual cancer (R1) | R0 : R1 | 20 : 3 | 24 : 2 | 28 : 7 | 0.376 |
